# A new species of the genus *Noeetomima* Enderlein (Diptera, Lauxaniidae) from Guizhou, China with a key to worldwide species

**DOI:** 10.3897/zookeys.1000.57577

**Published:** 2020-12-07

**Authors:** Li Shi, Miao Liu, Zheng-Kun Hu

**Affiliations:** 1 College of Horticulture and Plant Protection, Inner Mongolia Agricultural University, Hohhot 010018, China; 2 Administration of Fanjingshan National Nature Reserve, Guizhou 554400, China

**Keywords:** Fanjingshan National Nature Reserve, identification key, morphology, taxonomy, true flies

## Abstract

A species from the Fanjingshan National Nature Reserve in Guizhou Province, China is described as new to science: *Noeetomima huzhengkuni***sp. nov.** A key to separate worldwide species of *Noeetomima*, and a list of all species in the genus together with type information, is presented. The habitat of the new species is discussed.

## Introduction

The genus *Noeetomima* Enderlein, 1937 (Diptera, Lauxaniidae) was described for the new species *N. radiata* Enderlein, 1937 from Charbin (=Harbin, Heilongjiang Province, in northeast China). The next reference to this genus and species was in the key of Stuckenberg (1971a), with comments about the morphology of two new species that he ultimately described in Stuckenberg (1971b), namely *N. parva* Stuckenberg and *N. nepalensis* Stuckenberg from Queensland, Australia and Nepal, respectively. For *N. nepalensis*, a figure of the wing was presented in both Stuckenberg papers (1971a, 1971b), with figures of the wing for the other two species in Stuckenberg (1971b), where he also presented figures of the heads of the two new species, and male genitalia for *N. parva*. *Noeetomima nepalensis* was known only from the female in Stuckenberg (1971b), but Gaimari (in Shi et al. 2013) found a male from India. Stuckenberg (1971a) also presented lateral and dorsal views of the head of *N. radiata*. For *N. thaiensis*, Sasakawa (1987) described it from the female and provided a figure of the wing, and Gaimari (in Shi et al. 2013) illustrated the male genitalia. Kim (1994) treated the Australian species of the genus, providing figures of both male and female genitalia for *N. parva* and his new species *N. decora* Kim, and habitus and head illustrations for *N. decora*. Kim (1994) also expanded the known distribution of *N. parva* to include other states in eastern Australia. Shatalkin (1992) treated the Palaearctic species, recording *N. radiata* from the Russian Far East, and describing two new species from Kunashir Island in the (disputed) southern Kuril Islands, namely *N. aberrans* Shatalkin and *N. fulgens* Shatalkin, the former of which was described from the female only. Shatalkin (1992) provided figures of the wings for the three species he treated, but gave no genitalic illustrations. Shatalkin (2000) gave a key (translated into English by Schacht et al. (2004)) for the three Palaearctic species, repeating the wing figures from 1992, and providing a male genitalic illustration for *N. radiata*. Shi et al. (2013) described and illustrated *N. chinensis*, *N. tengchongica* and *N. yunnanica* from China, *N. jinpingensis* from China and Nepal with a key to 11 known species, and only recorded *N. aberrans* Shatalkin from Japan (no genitalic illustration), and proposed that the Australian species are different from the others in the antennal 1^st^ flagellomere being rounded at the tip, the abdomen being bicolored, the epandrium without a lateral split, and the surstylus having a single process. Li et al. (2020) described *N. hongshanensis*, *N. lijiangensis*, *N. liui* (only male with broken antennae), *N. trisurstyla* (male only) and *N. zhangae* (male only) with a key to the 16 known species.

The first author to consider the subfamily placement of *Noeetomima* was Stuckenberg (1971a), who placed the genus in the Lauxaniinae. The Oriental and Palaearctic catalogs (Shewell 1977; Papp 1984) continued with this placement, as did Sasakawa (1987), but Evenhuis and Okadome (1989) treated the genus in the Homoneurinae, which was followed by Kim (1994). Shatalkin (1992, 2000) gave no comments regarding subfamily placement, but Papp and Shatalkin (1998) treated it as Lauxaniinae in the key to genera. Kim (1994) included the genus in a phenetic analysis finding it close to *Trypetisoma* Malloch (which is itself only dubiously a homoneurine), which may be phylogenetically accurate, because the genera share an otherwise unique characteristic within the family – presence of a second anepisternal seta down-sloping and located near the middle of the anepisternum. Stuckenberg (1971a) noted this in his key to genera, but did not make any implication that he thought this genus was related to *Trypetisoma*, even though he extensively discussed Trypetisoma in his section on suprageneric classification. In that work, Stuckenberg (1971a) described the subfamily Homoneurinae, but explicitly excluded the genera *Trypetisoma* and *Trypaneoides* Tonnoir & Malloch (which is currently considered as a subgenus, or junior synonym, or *Trypetisoma*), despite many (but not all) species having his defining characteristic homoneuriform wing. Pending further work on *Trypetisoma* and related genera, Shi et al. (2013) consider *Noeetomima* to be in the Lauxaniinae because *Trypetisoma* and *Trypaneoides* were explicitly excluded from the Homoneurinae by Stuckenberg (1971a). Li et al. (2019) consider *Noeetomima* belongs to Lauxaniinae based on his research on *Trypetisoma*.

So far, there are 16 known species worldwide (Sasakawa 1987; Shatalkin 1992; Kim 1994; Shi et al. 2013; Li et al. 2020). The key to Old World genera presented in Stuckenberg (1971a), and the regional keys presented in Kim (1994), Papp and Shatalkin (1998) and Shatalkin (2000) (the latter of which is translated into English by Schacht et al. (2004)), worked to identify specimens to this genus, and Shi et al. (2013) gave a key to 11 known species, and Li et al. (2020) provided a key to the 16 known species.

In the present paper, a species new to science, *Noeetomima huzhengkuni* sp. nov., was collected from the Fanjingshan National Nature Reserve. The habitat of this new species is reported. A key to the known world species is presented along with a list of species in the Appendix 1.

## Material and methods

General terminology follows Cumming and Wood (2009), Gaimari and Silva (2010), and Shi and Yang (2014). Genitalia preparations were made by removing and macerating the apical portion of the abdomen in warm lactic acid for 10–20 minutes, then rinsing them with distilled water for dissection and study. After examination in glycerine, genitalia were transferred and stored in a microvial with glycerine pinned below the paratype.

Specimens were examined with a Nikon SMZ 1500 dissection microscope. Adult images were taken with a Nikon DS-Fi2 digital camera and a series of images montaged using Helicon Focus (HeliconSoft). All images and drawings were further processed with Adobe Photoshop CS 6.0.

The type specimens of the new species are deposited in the Insect Collection of Inner Mongolia Agricultural University, Hohhot, Inner Mongolia, China (IMAU).

## Taxonomy

### Noeetomima

Taxon classificationAnimaliaDipteraLauxaniidae

Enderlein

721B7D9A-BC29-5EE3-AD86-8521CE385B94

Noeetomima Enderlein, 1937: 73. Type species: Noeetomima radiata Enderlein (original designation). Stuckenberg 1971a: 559 (in key); Stuckenberg 1971b: 21 (diagnosis); Shewell 1977: 190 (catalog entry); Papp 1984: 203 (catalog entry); Evenhuis and Okadome 1989: 588 (catalog entry); Kim 1994: 22 (in key), 336 (diagnosis), 337 (key to Australian species); Papp and Shatalkin 1998: 395 (in key); Shatalkin 2000: 22 (in key), 35 (diagnosis, key to Palaearctic species); Schacht et al. 2004: 49 (in key), 57 (key to Palaearctic species); Shi et al. 2013: 340 (key to world species); Li et al. 2020: 500 (key to world species).

#### Diagnosis.

The genus can be easily identified by the wing patterning, which is dark centrally, with radiating hyaline stripes from the costal margin around to the posterior margin, and the posterior wing margin undulating between veins. Face yellow, with a pale brown subbasal and/or median band or spot and a pale brown groove near ventral margin; facial keel sometimes distinct or absent. Frons wider than long, with a pair of narrow brownish median stripes, parallel on anterior 1/2 and widened on posterior 1/2; two fronto-orbital setae, each with a blackish brown basal spot, two basal spots conjoined and forming a narrow stripe (not conjoined in *N. tengchongica* and *N. decora*); ocellar triangle grayish black. Antennal 1^st^ flagellomere tapering (round in *N. parva* and *N. decora*); arista white or black, pubescent; ocellar seta strong, longer than anterior fronto-orbital seta. Mesonotum with 1+3 dorsocentral setae and 1+3 acrostichal setae (including prescutellar) in 1–2 rows, each dorsocentral and acrostichal seta situated on a brown basal spot. 1 strong anepisternal seta, and second anepisternal seta down-sloping and located near the middle of the anepisternum; 2 strong katepisternal setae. Scutellum slightly convex with dense microtrichia. Fore femur without ctenidium; hind femur with 1–3 strong anteroventral setae (absent in *N. decora*, 4 in *N. huzhengkuni* sp. nov.). Wing mostly brown with hyaline or white spots and radiating stripes from costal margin around to posterior margin; posterior wing margin undulating between veins; cells r_2+3_ and r_4+5_ wide apically; a short apical section of R_2+3_ bent forwards and apical section of M_1_ obviously arched. Abdominal tergites with grayish white, brown or fulvous spots and long setae on posterior margin. Male genitalia: epandrium with rows of dorsal setulae and setae in posterior view and surstylus consisting of one (in *N. parva* and *N. decora*) or two processes (except for unknown male of *N. aberrans* and no male genitalic illustration of *N. fulgens*). If the surstylus is comprised of two processes which are separated by a deep trench generally, then there are many setae and setulae on the anterior process.

### Key to the known species of the genus *Noeetomima* worldwide

(Modified from Shi et al. 2013 and Li et al. 2020)

**Table:** 

1	Wing with a small brown central area, occupying 1/3 length of wing and several white radiating longest stripes between R_2+3_ and M_1_ longer than 1/2–2/3 length of ultimate sections of M_1_ (figs 2, 3 in Shi et al. 2013)	**2**
–	Wing with a large brown central area, occupying 2/3 length of wing and several white radiating longest stripes between R_2+3_ and M_1_ shorter than or close to 1/2 length of ultimate sections of M_1_ (Figs 7, 15)	**6**
2	Mesonotum with presutural dorsocentral and acrostichal setae at same horizontal level; scutellum shining without spot; wing with a suboval spot near middle	***N. radiata* Enderlein**
–	Mesonotum with presutural dorsocentral seta before horizontal level of presutural acrostichal seta; scutellum with a small grayish white tapering median spot, a pair of black lateral spots on basal 1/3 or 2/3 and a pair of grayish white round spots at base of basal scutellar seta; wing with a linear spot near middle	**3**
3	Wing with a white elliptical or quadrate spot present before vertical level of r-m in cell r_2+3_; hind femur with 1 strong anteroventral seta	**4**
–	Wing with a narrow stripe present before vertical level of r-m in cell r_2+3_; hind femur with 2–3 strong anteroventral setae	**5**
4	Fore femur with 3–4 strong posteroventral setae; surstylus consisting of a blunt triangular anterior process with setulae and a broad posterior digitiform process in lateral view	***N. jinpingensis* Shi, Gaimari & Yang**
–	Fore femur with 5 strong posteroventral setae; surstylus consisting of a long grayish black anterior ventral process with setulae and a short yellow rectangular process with a small brown horn–like process on anterior corner in lateral view	***N. tengchongica* Shi, Gaimari & Yang**
5	Hind femur with 2 strong anteroventral setae; mesonotum with a pair of black triangular posterior marginal spots extending to scutellum; surstylus with a posterior process broaden apically and truncated in lateral view	***N. liui* Li, Chen & Yang**
–	Hind femur with 3 strong anteroventral setae; mesonotum without pair of black triangular posterior marginal spots extending to scutellum; surstylus with 2 spiny posterior processes in posterior view	***N. trisurstyla* Li, Chen & Yang**
6	Arista white or pale yellow; wing with strongly undulating posterior margin	**7**
–	Arista dark brown or black; wing with only weakly undulating posterior margin	**11**
7	Hind femur with 1 strong anteroventral seta	***N. thaiensis* Sasakawa**
–	Hind femur with 2–3 strong anteroventral setae	**8**
8	Wing with three white spoon-like spots present along costal margin; surstylus consisting of a long claviform anterior process with setulae and a furcated digitiform posterior apical process in lateral view	***N. yunnanica* Shi, Gaimari & Yang**
–	Wing with a row of white triangular spots present along costal margin; surstylus not as above in lateral view	**9**
9	Eye with a concavity on posterior ventral margin; surstylus consisting of a long claviform anterior process with setulae and a short digitiform posterior apical process in lateral view	***N. nepalensis* Stuckenberg**
–	Eye with straight posterior ventral margin; surstylus not as above	**10**
10	Face with a pale brown V-shaped median spot; apical half of wing with three long linear spots connecting R_2+3_ with R_4+5_ in cell r_2+3_; surstylus consisting of a long furcated anterior process and a posterior apical process without median process on anterior margin in lateral view	***N. chinensis* Shi, Gaimari & Yang**
–	Face without pale brown V-shaped median spot; apical half of wing with several short linear spots along lower margin of R_2+3_ and upper margin of R_4+5_ in cell r_2+3_; surstylus consisting of an anterior process tapering apically and a posteroapical process with 1 median process on anterior margin in lateral view	***N. zhangae* Li, Chen & Yang**
11	Antennal 1^st^ flagellomere tapering distally; abdominal tergites 1–6 unicolorous, only dark brown	**12**
–	Antennal 1^st^ flagellomere rounded at tip; abdominal tergites 1–6 bicolored, at least tergites 1–3 fulvous and tergites 5–6 blackish brown	**16**
12	Mesonotum with presutural dorsocentral and acrostichal setae at same horizontal level; wing with brown irregular spots and white spots near bottom of R_4+5_	***N. aberrans* Shatalkin**
–	Mesonotum with presutural dorsocentral seta before horizontal level of presutural acrostichal seta; wing with brown regular spots and white radiating stripes near bottom of R_4+5_	**13**
13	Mid and hind femora in basal 3/4 darkened	**14**
–	Mid and hind femora completely yellow	**15**
14	Hind femur with 4 strong anteroventral setae (3 very strong) on apical half (Fig. 1); wing with white spots arranged in a triangle in cell r_2+3_ (Figs 7, 15); surstylus with wide truncate posterior process with a concavity at anterior corner in lateral view and short dense setulae at apex in posterior view (Figs 16, 17)	***N. huzhengkuni* sp. nov.**
–	Hind femur with 2 strong anteroventral setae; wing with white spots situated in a straight line in cell r_2+3_; surstylus with posterior process broaden apically and concaved medially in lateral view, but no short dense setulae at apex in posterior view	***N. hongshanensis* Li, Chen & Yang**
15	Mid and hind tibiae yellow with a brown ring in basal 1/3; wing with 3 tiny white round spots situated in cell r_2+3_ and 6 white radiating stripes along margin between tips of R_2+3_ and M_1_	***N. lijiangensis* Li, Chen & Yang**
–	Mid and hind tibiae in basal 3/4 black; wing with 2 bigger white irregular spots situated in cell r_2+3_ and 4–5 white radiating stripes along margin between tips of R_2+3_ and M_1_	***N. fulgens* Shatalkin**
16	Wing with series of longitudinal white lines through cells r_2+3_ and r_4+5_; hind femur with 1 strong anteroventral seta	***N. parva* Stuckenberg**
–	Wing with few spots and paler brown patches in cells r_2+3_ and r_4+5_, but no linear spots; hind femur without strong anteroventral setae but with strong black spinules	***N. decora* Kim**

### Noeetomima huzhengkuni

Taxon classificationAnimaliaDipteraLauxaniidae

Shi & Liu sp. nov.

60631A96-9467-5AC7-8CAE-6F284C6B66B8

http://zoobank.org/1B43FC68-840D-413F-AF50-F8A01D1EE6C2

Figs 1–7 , 8–15 , 16–20

#### Type material.

***Holotype*** ♂ (IMAU): China, Guizhou Province: Fanjingshan, primitive forest along cableway station entrance to Wanbaoyan scenic spot, elevation 1800–2200 m, 27°56'N, 108°46'E, 06. vi. 2018, Zheng-Kun Hu. ***Paratype***: 1♂ (IMAU), data same as holotype except for male genitalia being dissected.

#### Diagnosis.

**Male.** Arista brown. Palpus dark brown. Mesonotum with presuturaldorsocentral seta before horizontal level of presutural acrostichal seta. Fore femur with 3 strong posteroventral setae and hind femur with 4 anteroventral setae (3 very strong) on apical half. Wing with large brown central area, occupying 5/6 length of wing and several white radiating stripes shorter than 1/2 length of ultimate section of M_1_, two smaller white round spots in cell r_2+3_ and a bigger one in cell r_4+5_ situated in a straight line on 1/5 length of wing. Male genitalia: syntergosternite 7+8 pale brown and epandrium brownish yellow; surstylus consisting of a long anterior process with 8–10 long setae on dorsal margin and a short wide truncate posterior apical process with a concavity at anterior corner in lateral view and short dense setulae at apex in posterior view.

**Figures 1–7.**
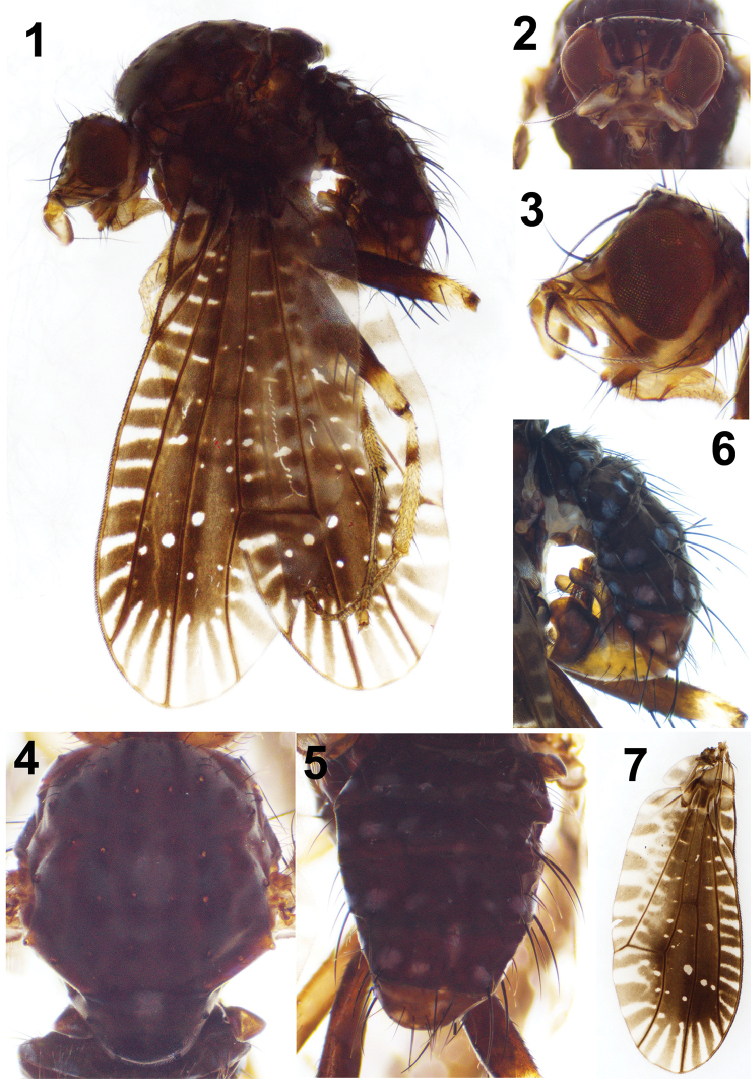
*Noeetomima huzhengkuni* sp. nov. Holotype male **1** habitus, lateral view **2, 3** head, anterior and lateral view **4** thorax, dorsal view **5, 6** abdomen dorsal and lateral view **7** wing.

#### Description.

**Male.** Body length 2.8–2.9 mm, wing length 4.3–4.5 mm.

***Head*** (Figs 2–3, 9–10) pale yellow. Face with a brown median spot near ventral margin, a pale-yellow groove above ventral margin; parafacial pale yellow except blackish brown inner margin, with narrow brown median stripe on dorsal 1/2 and an elliptical brown ventroapical spot. Frons dark brown on dorsal 1/3 (from two sides of ocellar triangle to vertex), a pair of brownish median stripes extending from anterior margin to ocellar triangle and a pair of brown lateral stripes along base of fronto-orbital setae extending to vertex. Ocellar triangle dark brown. Gena with blackish brown spot near posterior ventral margin of eye, about 1/4 height of eye. Occiput dark brown with grayish pruinosity. Antennal scape and pedicel pale brown, 1^st^ flagellomere pale brown on dorsal and ventral margin, completely brown near base of arista, about 1.6 times longer than high; arista brown, pubescent. A brown stripe present between eye and antenna. Eye without concavity on posterior ventral margin. Clypeus brown. Proboscis pale yellow, palpus dark brown.

***Thorax*** (Figs 4, 11, 13) brown, with thick gray pruinosity. Mesonotum with pair of narrow brown median stripes extending from anterior margin to presutural acrostichal seta, posterior margin of postpronotum surrounded by brown irregular spots, 3–4 brown irregular spots scattered between dorsocentral and supraalar setal rows, strong setae of mesonotum each with a brown basal spot; presutural dorsocentral seta before horizontal level of presutural acrostichal seta, postsutural 1^st^ dorsocentral seta near transverse scutal suture and postsutural 1^st^ acrostichal seta on transverse scutal suture; prescutellar acrostichal setae shorter than 1^st^ postsutural dorsocentral setae. Anepisternum and katepisternum brown with grayish pruinosity; anepisternum with two brown spots on upper half and a brown spot on lower margin, 2 anepisternal setae separately located on posterior margin and close to anterior margin; katepisternum with a yellow transverse stripe close to upper margin and brown base of 2 katepisternal setae. Scutellum shining blackish brown, with grayish white pruinosity on basal 1/4–1/3 and dense microtrichia on apical 1/2, and a yellow central spot on upper half and upper lateral margin of basal scutellar setae brownish yellow. Legs pale yellow, fore femur pale brown on basal 3/4, mid and hind femora dark brown on basal 3/4; fore tibia with a pale brown subbasal ring, mid and hind tibae each with a dark brown subbasal ring; all tarsomeres 4–5 pale brown. Fore femur with 3 strong posteroventral setae and 5–6 posterodorsal setae; fore tibia with 1 long preapical anterodorsal seta and 1 short apicoventral seta. Mid femur with 3 anterior setae on apical half and 1 apicoposterior seta; mid tibia with 1 preapical anterodorsal seta and 1 apicoventral seta. Hind femur with 4 anteroventral setae on apical half (3 very strong) and 1 preapical anterodorsal seta; hind tibia with 1 preapical anterodorsal seta and 1 short apicoventral seta. *Wing* (Figs 7, 15) with large brown central area, occupying 5/6 length of wing and several white radiating stripes shorter than 1/2 length of ultimate section of M_1_, seven white radiating stripes along margin between tips of R_2+3_ and CuA_1_; two smaller white round spots in cell r_2+3_ and a bigger one in cell r_4+5_ situated in a straight line on 1/5 length of wing; posterior margin slightly undulating; costa with 2^nd^ (between R_1_ and R_2+3_), 3^rd^ (between R_2+3_ and R_4+5_) and 4^th^ (between R_4+5_ and M_1_) sections in proportion of 3.1 : 1.2 : 1; *r-m* beyond middle of discal cell; ultimate and penultimate sections of M_1_ in proportion of 1 : 1.7; ultimate section of CuA_1_ about 1/11 of penultimate. Halter pale yellow, except knob brown.

**Figures 8–15.**
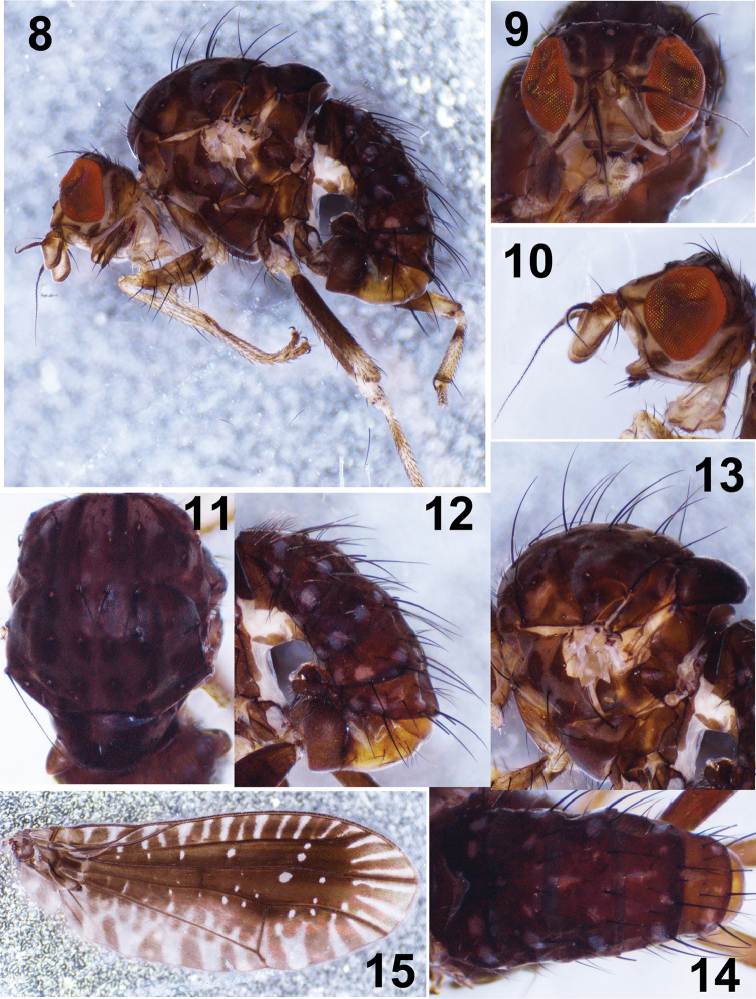
*Noeetomima huzhengkuni* sp. nov. Paratype male **8** habitus, lateral view **9, 10** head, anterior and lateral view **11, 13** thorax, dorsal and lateral view **12, 14** abdomen lateral and dorsal view **15** wing.

**Figures 16–20.**
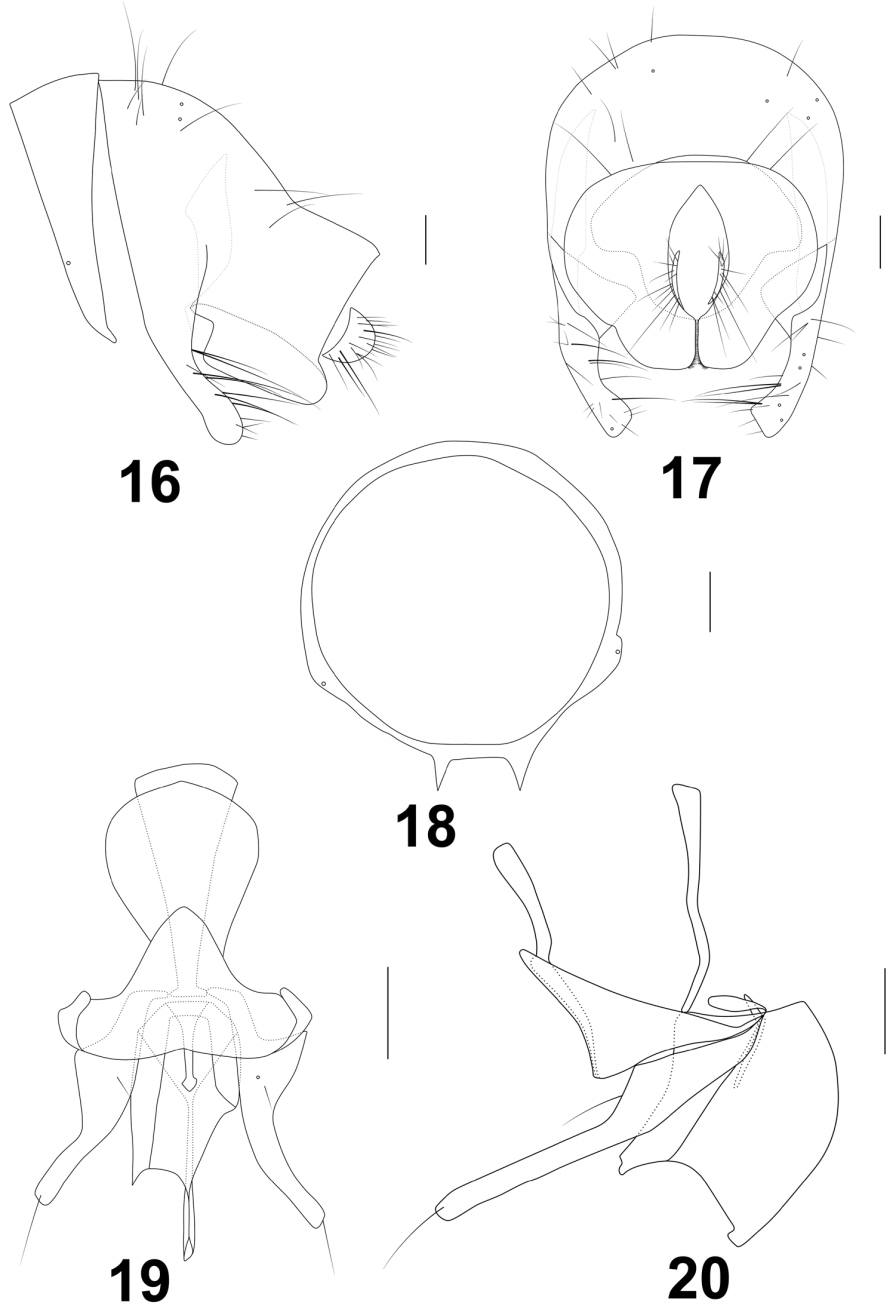
*Noeetomima huzhengkuni* sp. nov. Paratype male **16** syntergosternite and epandrium, lateral view **17** epandrial complex, posterior view **18** syntergosternite, anterior view **19** phallic complex, ventral view **20** phallic complex, lateral view. Scale bars: 0.1 mm.

***Abdomen*** (Figs 5–6, 12, 14) dark brown with grayish yellow pruinosity. Tergites 3–6 each with grayish white median spot on anterior margin, tergites 2–6 each with 8 grayish white spots and 4 pairs of setae on posterior margin. *Male genitalia* (Figs 16–20): syntergosternite 7+8 pale brown and epandrium brownish yellow; syntergosternite 7+8 slender, circular with a pair of ventral processes; epandrium broaden with 6 rows of dorsal setae, each with brownish basal spot; surstylus consisting of long anterior process with 8–10 long setae on dorsal margin and short wide truncate posterior apical process with a concavity at anterior corner in lateral view and short dense setulae at apex in posterior view; hypandrium broad, nearly T–shaped; pregonite long with a basal setula, curved medially and narrow apically with a long setula; phallus broad and concaved at apex in lateral view, but wide basal 2/3 with an arrow-like basal process, slender apical 1/3 and lateral sclerites asymmetric in ventral view. Phallapodeme longer than hypandrium in lateral view. Cerci very small.

**Female.** Unknown.

#### Etymology.

The species is named after the collector and amateur of insects Zheng-Kun Hu.

#### Distribution.

China (Guizhou).

#### Remarks.

In the present paper, the first author divides 17 known species into two groups: the *N. radiata*-group includes *Noeetomina liui*, *N. jinpingensis*, *N. radiata*, *N. tengchongica* and *N. trisurstyla*, which several white radiating longest stripes between R_2+3_ and M_1_ are longer than 1/2–2/3 length of ultimate sections of M_1_ on the wing; the *N. parva*-group includes *Noeetomima aberrans*, *N. chinensis*, *N. decora*, *N. fulgens*, *N. hongshanensis*, *N. huzhengkuni* sp. nov., *N. lijiangensis*, *N. nepalensis*, *N. parva*, *N. thainensis*, *N. yunnanica*, *N. zhangae*, which several white radiating longest stripes between R_2+3_ and M_1_ are shorter than or close to 1/2 length of ultimate sections of M_1_.

Compared to five species in the *N. radiata*-group, the new species differs by the length of white radiating longest stripes between R_2+3_ and M_1_ on the wing and the number of anteroventral setae on apical half on hind femur, but the mid and hind femora have same dark brown on basal 3/4 in *Noeetomima liui*, *N. jinpingensis*, *N. tengchongica* as the new species.

Compared to the other eleven species in the *N. parva*-group, the new species differs from *N. chinensis*, *N. decora*, *N. hongshanensis*, *N. lijiangensis*, *N. nepalensis*, *N. parva*, *N. thainensis*, *N. yunnanica* and *N. zhangae* by the following two features: the mid and hind femora are completely dark brown on the basal 3/4; the phallus is broad and curved at apex in lateral view, but it is wide in the basal 2/3 with an arrow-like basal process and is slender in the apical 1/3, and the lateral sclerites are asymmetric in ventral view.

*Noeetomima aberrans*, with unknown male, can be separated from the new species in the katepisternum having a parallel pair of gray stripes on the upper margin and slightly below, the gray scutellum having a pair of large brownish spots (sometimes fusing at the apex), four white radiating longest stripes between R_2+3_ and M_1_ being shorter than 1/2 length of the ultimate sections of M_1_ on the wide wing, and the presutural dorsocentral setae being the same level as the presutural acrostichal setae on the mesonotum.

*Noeetomima fulgens*, without a male genitalic illustration, can be separated from the new species in the spots at the base of the presutural dorsocentral setae being very small on the mesonotum, a pair of narrow middle stripes on the mesonotum being not developed beyond suture, the anepisternum being brown with a gray stripe extending from the anterior upper comer to the lower posterior corner, the katepisternum having a large brownish spot in the anterior part and a narrow brownish stripe surrounding the bases of the katepisternal setae, the scutellum having densely gray microtomentose on the dorsal side and wide brown margins, the mid and hind tibiae being black in the basal 3/4, the fore femora being darkened only dorsally and laterally in the basal 3/4, the wing having five white radiating longest stripes between R_2+3_ and M_1_ close to or shorter than 1/2 length of the ultimate sections of M_1_, and a big hyaline elliptical spot being present before the vertical level of crossvein r-m in r_2+3_, and a big hyaline round spot being present before the vertical level of crossvein r-m in discal cell.

The new species from Guizhou is so similar to *Noeetomima lijiangensis* Li, Chen & Yang from Sichuan and Yunnan of China in the anterior projection of the frons and the face, and the pattern of mesonotum, scutellum and wing, but the latter can be separated in the abdominal tergites 4–6 having broad white spots (narrow stripes in *N. huzhengkuni* sp. nov.), the epandrium being flat in the anterior half (having an obvious bulge in the anterior half in *N. huzhengkuni* sp. nov.), the anterior process of the surstylus having short setulae and a small median anterior projection in lateral view (having long setae, but no small median anterior projection in *N. huzhengkuni* sp. nov.), the posterior process of the surstylus having a preapical anterior projection in lateral view and narrowing gradually at the apex (broadened apically with a deep concavity at the anterior corner in lateral view and having short dense setulae at the apex in posterior view in *N. huzhengkuni* sp. nov.), the pregonite being obviously shorter than the length of phallus in lateral view (both equal in length in *N. huzhengkuni* sp. nov.), the phallus having pairs of teeth-like processes near the base and apical in ventral view with the apex curved dorsally in lateral view (being broad and concaved at the apex in lateral view, but being wide basal 2/3 with an arrow-like basal process, slender apical 1/3 and lateral sclerites asymmetric in ventral view in *N. huzhengkuni* sp. nov.), the phallapodeme being as long as 1/2 length of phallus in lateral view (both equal in length in *N. huzhengkuni* sp. nov.).

### Habitat and plant

Fig. 21

The Fanjingshan National Nature Reserve is located in the northeast of Guizhou Province, China with the geographical coordinates between 27°49"50' to 28°1"30'N and 108°45'55" to 108°48'30"E . The Fanjingshan area is a monsoon climate region of East Asia, which has typical characteristics of a humid climate in a mid subtropical monsoon mountain. It is a well-preserved natural primitive complex with scientific research value. There are more than 2000 species of insects.

The individuals of this species are active on sunny days and more often at noon in the Fanjingshan National Nature Reserve. They are easily and quickly captured when approached slowly. For the behavior of this species, the collector Zheng-Kun Hu did not observe courtship or mating or competition for food between individuals, which never moved quickly to forage. They prefer to gather on the rocks or stones in the shade or two or three will stop on broad fleshy leaves or bamboo leaves in the sun, often motionless, or only with the wings moving slowly up and down; they probably feed on fungi on the surface of rocks and leaves. They prefer to move slowly or stop on broad fleshy leaves such as those of the genera *Ligularia* Cass (Asteraceae), *Strobilanthes* Blume (Acanthaceae), *Impatiens* L. (Balsaminaceae), *Reineckia* Kunth (Asparagaceae) and various species of Polygonaceae. Its habitat is mainly primitive forest, which is comprised of the dominant tree species *Quercus multinervis* (Cheng WC and Hong T) Li JQ (Fagaceae), the dominant bamboo species *Fargesia spathacea* Franch. (Poaceae) and various perennial herbaceous plants under the forest canopy.

**Figure 21.**
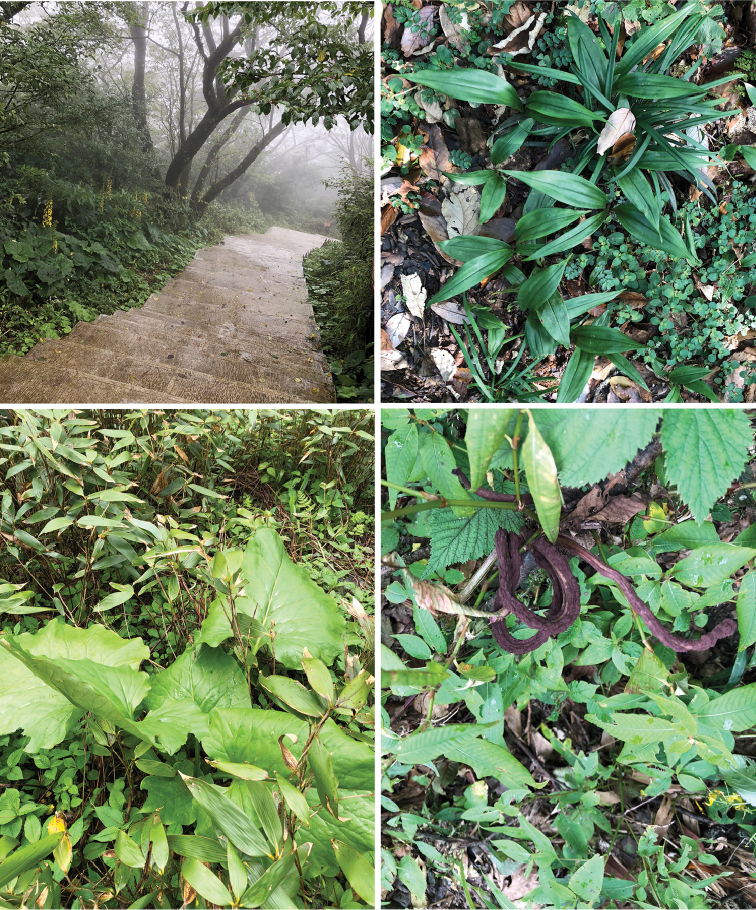
Habitat and plants where *Noeetomima huzhengkuni* sp. nov. is found.

## Supplementary Material

XML Treatment for Noeetomima

XML Treatment for Noeetomima huzhengkuni

## Appendix 1

**Species list of the genus *Noeetomima* in the world**

1. Noeetomima aberrans Shatalkin, 1992: 79. Type locality: Russia (disputed), Kuril Islands, Kunashir Island, near Mendeleev volcano. Type: holotype ♀ (ZMUM). Palaearctic Region: Japan (Hokkaido), Russia (South Kuril Islands).
2. Noeetomima chinensis Shi, Gaimari & Yang, 2013: 341. Type locality: China, Zhejiang Province, Longquan, Fengyangshan National Nature Reserve, Huangmaojian. Type: holotype ♂ (CAUC). Oriental Region: China (Guizhou, Zhejiang).
3. Noeetomima decora Kim, 1994: 337. Type locality: Australia, New South Wales, Coffs Harbour. Type: holotype ♂ (ANIC). Australian Region: Australia (New South Wales, Queensland).
4. Noeetomima fulgensShatalkin 1992: 81. Type locality: Russia (disputed), Kuril Islands, Kunashir Island, near Mendeleev volcano. Type: holotype ♂ (ZMUM). Palaearctic Region: Russia (South Kuril Islands).
5. Noeetomima hongshanensis Li, Chen & Yang, 2020. Type locality: China, Yunnan Province, Xiaozhongdian Hongshan forestry farm. Type: holotype ♂ (CAUC). Oriental Region: China (Sichuan, Yunnan).
6. Noeetomima huzhengkuni sp. nov. Type locality: China, Guizhou Province, Fanjingshan National Nature Reserve. Type: holotype ♂ (IMAU). Oriental Region: China (Guizhou).
7. Noeetomima jinpingensis Shi, Gaimari & Yang, 2013. Type locality: China, Yunnan Province, Jinping, Yakou. Type: holotype ♂ (CAUC). Oriental Region: China (Yunnan), Nepal (Arun Valley).
8. Noeetomima lijiangensis Li, Chen & Yang, 2020. Type locality: China, Yunnan Province, Lijiang mountain botanical garden. Type: holotype ♂ (CAUC). Oriental Region: China (Sichuan, Yunnan).
9. Noeetomima liui Li, Chen & Yang, 2020. Type locality: China, Yunnan Province, Tengchong Shabacun. Type: holotype ♂ (CAUC). Oriental Region: China (Yunnan).
10. Noeetomima nepalensis Stuckenberg, 1971: 24. Type locality: Nepal, Ulleri. Type: holotype ♀ (BMNH). Oriental Region: India (Meghalaya), Nepal (Sikha, Ulleri).
11. Noeetomima parva Stuckenberg, 1971: 27. Type locality: Australia, Queensland, Brisbane. Type: holotype ♀ (AMSA) [note, Stuckenberg (1971) indicated University of Queensland]. Australian Region: Australia (Australian Capital Territory, New South Wales, Queensland).
12. Noeetomima radiata Enderlein, 1937: 73. Type locality: Charbin, Manchuria [=Harbin, Heilongjiang Province, China]. Type: holotype ♂ (ZMHB). Palaearctic Region: China (Heilongjiang), Russia (Primorye and Khabarovsk Region).
13. Noeetomima tengchongica Shi, Gaimari & Yang, 2013. Type locality: China, Yunnan Province, Tengchong, Tengchong Village. Type: holotype ♂ (CAUC). Oriental Region: China (Yunnan).
14. Noeetomima thaiensis Sasakawa, 1987: 1. Type locality: Thailand, Chiang Mai, Doi Inthanon. Type: holotype ♀ (UOPJ). Oriental Region: Thailand.
15. Noeetomima trisurstyla Li, Chen & Yang, 2020. Type locality: China, Guangxi Province, Nanning Damingshan. Type: holotype ♂ (CAUC). Oriental Region: China (Guangxi).
16. Noeetomima yunnanica Shi, Gaimari & Yang, 2013. Type locality: China, Yunnan Province, Jinping, Fenshuiling Protection Station. Type: holotype ♂ (CAUC). Oriental Region: China (Yunnan).
17. Noeetomima zhangae Li, Chen & Yang, 2020. Type locality: China, Yunnan Province, Jinping yakou. Type: holotype ♂ (CAUC). Oriental Region: China (Yunnan).
